# A Novel Adaptive Synchrosqueezing Transform Based on Enhanced STFT Scheme for Wind Turbine Bearing Fault Diagnosis

**DOI:** 10.3390/s26206564

**Published:** 2026-10-16

**Authors:** Zhexi Huang, Cancan Yi, Xuechong Fang, Fanzhou Yang, Dongwei Deng

**Affiliations:** 1 Key Laboratory of Metallurgical Equipment and Control Technology, Ministry of Education, Wuhan University of Science and Technology, Wuhan 430081, China; 2 Wenzhou Special Equipment Inspection & Science Research Institute, Wenzhou 325000, China; 3 Hubei Energy Group New Energy Development Co., Ltd., Wuhan 430077, China

**Keywords:** wind turbines, fault diagnosis, Short-Time Fourier Transform, time–frequency analysis, adaptive Synchrosqueezing algorithm, instantaneous frequency estimation

## Abstract

Accurate estimation of the instantaneous frequency (IF) of multi-component non-stationary signals is crucial for the fault diagnosis of wind turbine transmission systems under variable speed. Current methodologies exhibit several key limitations, such as unsatisfied accuracy of instantaneous parameter estimation due to the inadequate time–frequency representation, poor time–frequency energy concentration and multimode separation effects caused by a single post-processing technique, and the inability of non-adaptative window parameters to dynamically accommodate the local time-varying frequency characteristics of signals. These deficiencies degrade time–frequency plane interpretability and the accuracy of extracting time-varying fault features. To address these challenges, this paper proposes a novel Improved Short-Time Fourier Transform-based Adaptive Synchrosqueezing Transform (ISTFT-ASST) and demonstrates its application in wind turbine bearing fault diagnosis. Firstly, to achieve the precisely parameter estimation of multi-component non-stationary signals, an improved Short-Time Fourier Transform (STFT) sparse observation model integrated with the Finite Rate of Innovation (FRI) is innovatively constructed. Secondly, Synchrosqueezing Extraction Transform (SET), a time–frequency post-processing technique, is incorporated to further enhance the time–frequency concentration. Finally, drawing on the idea of local Rényi entropy-based window parameter estimation, an adaptive Synchrosqueezing algorithm is designed to dynamically accommodate the time-varying frequency characteristics. Through the analysis of numerical simulation signals and measured wind turbine vibration signals, the effectiveness of the proposed ISTFT-ASST in extracting time-varying features is verified, establishing a novel approach for wind turbine fault diagnosis.

## 1. Introduction

Wind turbine fault diagnosis is vital to implement predictive maintenance, cut operating expenses and improve power generation stability. Under variable-speed working conditions, the generator transmission system (including the main shaft, gearbox and rolling bearings) generates complicated coupled vibrations, a typical feature widely acknowledged in wind turbine fault diagnosis research [1]. In variable-speed wind turbine drive systems, rolling bearing fault signals typically manifest as non-stationary amplitude-modulated and frequency-modulated (AM-FM) signals. The dynamic variation of IF directly reflects the progression patterns of faults, such as rolling bearing wear and spalling. Accurately capturing these dynamic parameters is central to early fault identification. At present, three prominent technical bottlenecks exist in wind turbine vibration signal processing. To begin with, incipient fault signatures are very faint and easily submerged by intense operational noise. Secondly, IF varies drastically with wind speed fluctuation and fault evolution. Such irregular variations greatly increase the difficulty of feature extraction. Thirdly, multi-component signals suffer severe mode aliasing, as fault characteristic ridges overlap with interference components, such as gear meshing harmonics and blade aerodynamic vibration signals. Therefore, accurately extracting IFs under variable-speed, high-noise, and multi-mode aliasing conditions is crucial for achieving early warning and localization of wind turbine bearing faults [2]. To address these challenges, this study therefore focuses on developing a real-time, adaptive time–frequency analysis method capable of precise instantaneous frequency estimation under such complex conditions.

Time–frequency analysis (TFA) provides the core framework for processing such non-stationary signals and directly determines the reliability of diagnostic outcomes. Research has evolved from classical time–frequency representations to adaptive post-processing strategies. Synchrosqueezing Wavelet Transform (SSWT), a core variant of the Synchrosqueezing Transform (SST), is widely applied in wind turbine bearing fault diagnosis [3]. This method redistributes time–frequency coefficients based on the Continuous Wavelet Transform (CWT), effectively addressing the inadequate time–frequency concentration inherent in conventional CWT and enhancing fault feature recognition [4]. Chirp Transform (CT) adapts to linearly chirped signals through frequency modulation analysis, enabling flexible time–frequency resolution adjustment to accommodate speed-induced frequency variations in wind turbine operations [5]. High-Order Synchrosqueezing Transform (HSST) enhances the focusing effect of time–frequency ridges via a higher-order redistribution mechanism, further addressing the resolution limitations of traditional time–frequency transforms [6]. Oberlin, Meignen, and Perrier [7] proposed the enhanced Synchrosqueezing transform, replacing first-order transient frequency estimation with a second-order strategy for improved accuracy. This effectively resolves the energy dispersion issue in TFA of strongly frequency-modulated signals. However, the aforementioned enhanced time–frequency transforms still suffer from critical limitations under variable-speed operating conditions. For instance, while SSWT enhances time–frequency concentration, it remains constrained by fixed wavelet bases. Fluctuations in turbine speed cause wide-range and high-rate frequency variations in signals, preventing wavelet bases from dynamically matching local frequency changes and often leading to time–frequency ridge diffusion [8]. CT exhibits poor adaptability to signals with strong frequency variations and suffers pronounced time–frequency aliasing in scenarios with multiple coupled components. HSST enhances time–frequency ridge focusing through higher-order redistribution but incurs significant computational overhead due to intensive operations. SET improves modal separation for moderately noisy signals, yet it lacks dedicated noise suppression and weak feature enhancement mechanisms tailored for time–frequency representations. When the signal-to-noise ratio drops, noise-related time–frequency coefficients cannot be filtered out, and faint fault components are easily submerged, resulting in degraded feature extraction performance under heavy noise interference. To resolve parameter non-adaptivity, researchers have further proposed adaptive Synchrosqueezing transform methods. Li Lin et al. [9] developed a local Rényi entropy-optimized adaptive Synchrosqueezing transform for better ridge matching of time-varying signals. Unfortunately, fixed neighborhood radius in its window optimization leads to adaptive delay under abrupt speed shifts, which reduces IF tracking accuracy and fails to meet the precision demand of incipient wind turbine fault detection.

In parallel, efforts have been devoted to optimizing the STFT architecture and its adaptive mechanisms. Xiaowei Li et al. [10] proposed an optical-domain channelized analog microwave STFT method, leveraging channelization techniques to optimize time–frequency analysis across different frequency bands and thereby improving processing adaptability for wideband signals. However, this approach fundamentally remains constrained by fixed-window non-stationary functions. The resulting time–frequency basis representation lacks sparsity, making instantaneous parameter estimation susceptible to interference from redundant information. Rao Xianjie et al. [11] proposed an improved scheme based on Short-Time Fractional Fourier Transform (STFrFT) by dynamically adjusting the transform rotation angle to suppress cross-terms, thereby improving time–frequency resolution for multi-component signals. Nevertheless, this method relies solely on a single-transform processing flow without integrating specialized time–frequency sharpening techniques, resulting in suboptimal energy aggregation and mode separation. Maxime Leiber et al. [12] introduced the Differentiable Adaptive STFT (DASTFT), dynamically optimizing window length via gradient descent to enhance window adaptation to local signal features. However, this approach only achieves unidimensional optimization of window length, lacking a multidimensional adaptation mechanism tailored to signal frequency variations. Consequently, its parameter adjustment flexibility and real-time performance remain inadequate. In summary, existing STFT improvements perform localized optimizations for isolated issues but fail to address fundamental limitations in time–frequency representations—specifically, excessive redundant spectral components, low feature discriminability, and misalignment between parameter adaptation and signal time-varying characteristics—leading to constrained overall diagnostic effectiveness. More recently, several TFA methods have been specifically developed for wind turbine fault diagnosis under non-stationary conditions, yet critical gaps persist. Wu et al. [13] proposed an Adaptive Matching Squeezing Chirplet Transform (AMSCT) that constructs a polynomial chirp rate regulated by a single parameter, coupled with a time–frequency concentration criterion, to extract closely spaced and crossed instantaneous frequencies in planetary gearboxes. While AMSCT demonstrates improved resolution for multi-component signals, its post-processing framework relies on bi-directional ridge detection and Vold–Kalman filtering, which introduces additional computational layers and manual parameter tuning, limiting its real-time applicability and robustness to abrupt speed variations. From a different perspective, Krishnendu et al. [14] proposed a Teager energy concentrated time–frequency transform (TSTPOW), in which the analysis window is optimized according to a Teager energy concentration criterion to produce sharply localized representations of transient bearing fault signatures. Although this method attains high energy concentration, its window optimization relies on iterative searching that incurs considerable computational cost, and the resulting time–frequency representation does not provide explicitly extracted, physically interpretable IF trajectories, which limits its mechanism-level interpretability and real-time applicability. These recent advances underscore a persistent trade-off between representation quality and practical deploy ability.

To address the aforementioned limitations, this paper introduces the FRI model as a cornerstone for sparse signal analysis. The model, a cornerstone of sparse signal processing, represents signals with a finite number of non-zero parameters as sparse Dirac combs. Leveraging signal sparsity, it enables precise extraction of feature parameters from redundant observations [15]. Its core advantage lies in effectively separating noise and redundant spectral components, enhancing parameter representation, and significantly improving the robustness and accuracy of parameter estimation in noisy, non-stationary scenarios. This theory provides a novel sparse modeling framework for TFA of non-stationary signals. By structurally integrating FRI with STFT, a sparse time–frequency observation model can be established, inherently enhancing representation sparsity and feature interpretability. Specifically, the conventional symmetric analysis window is replaced by a causal window tailored for FRI modeling, and the corresponding frequency-domain convolution kernel is derived. The STFT spectrum is thus reformulated as a convolution of sparse Dirac combs with the kernel, mapping instantaneous frequency and amplitude directly to the Dirac comb positions and weights.

Most existing Finite Rate of Innovation based Short-Time Fourier Transform (FRI-STFT) implementations rely on block-wise processing and iterative reconstruction to achieve sparsity. Although they can build sparse representations for non-stationary signals, these schemes are designed for general-purpose signal processing and lack modal-weighting post-processing, as well as entropy-guided adaptive window optimization. Under variable-speed conditions with heavy multi-mode aliasing, iterative block processing introduces tracking lag, thereby degrading estimation accuracy.

In contrast, the proposed ISTFT-ASST directly extracts IFs via a non-iterative sparse model and adapts window parameters in real time, thereby circumventing both the reliance on tachometer signals and the computational burden of post-processing pipelines. Its core innovations are as follows: (1) a novel STFT sparse observation model correlated with the FRI model is constructed, overcoming limitations in traditional STFT and existing adaptive window designs while fundamentally enhancing time–frequency representation quality and enabling precise instantaneous frequency estimation; (2) multi-model post-processing techniques from SET [16] are integrated to enhance spatiotemporal energy concentration, addressing insufficient energy focus under strong noise interference; (3) an adaptive Synchrosqueezing algorithm is designed based on local Rényi entropy-based window parameter estimation, dynamically adapting to local frequency variation rates and automatically adjusting compression parameters to overcome limitations in parameter adaptability and real-time capability. Crucially, the proposed ISTFT-ASST breaks through the real-time bottleneck that plagues existing adaptive TFA methods. Its implementation involves only fast Fourier transforms, windowing, and closed-form kernel operations, yielding a computational complexity of O (N log N) and a negligible memory footprint. To validate the proposed method, multi-scenario test datasets are constructed using numerical simulation signals and real-world wind turbine bearing fault signals. This paper aims to develop the ISTFT-ASST time–frequency analysis method for accurate extraction of wind turbine bearing fault features under variable-speed and strong-noise conditions. Furthermore, multi-dimensional validations are conducted through theoretical analysis, numerical simulation, laboratory bench experiments and field-measured data to achieve accurate fault-feature identification and reliable condition monitoring.

## 2. Theoretical Description

### 2.1. Sparse Observation Model of STFT

Generally, the discrete time-domain mathematical model of multi-component non-stationary signals is defined as a linear superposition of AM-FM signals, which is expressed as follows:

(1) $$s\left(n\right)=\sum_{k=1}^{K}a_{k}\left(n\right)e^{j\varphi_{k}\left(n\right)},\;n=0,1,\ldots ,N-1$$

where *n* ∈ {0,1,…,*N* − 1} is the time index, *N* is the total length of the signal, *K* represents the number of signal components, *α_k_*(*n*) ≥ 0 is the instantaneous amplitude (IA) of the *k*-th component, *φ_k_*(*n*) is the phase function whose time derivative *ω_k_*(*n*) = ∂*φ_k_*(*n*)/∂*n* corresponds to the IF.

The basic STFT conducts local time–frequency analysis via a sliding fixed window, defined as:

(2) $${\mathrm{STFT}}_{x}\left(n,m\right)=\sum_{l=-\infty }^{+\infty }s\left(l\right)w\left(n-l\right)e^{-\mathrm{jml}}$$

where *l* is the time shift, *m* ∈ {0,1,…,*M* − 1} is the discrete frequency index, and *e*^−*jml*^ is the Fourier transform basis function. Taking the squared modulus of the STFT result achieves the spectrogram as follows:

(3) $$P\left(n,m\right)={\left|{\mathrm{STFT}}_{x}\left(n,m\right)\right|}^{2}$$

However, constrained by the Heisenberg uncertainty principle, fixed windows fail to balance time–frequency resolution [17] and suffer from severe mode aliasing [18] for fast-varying multi-component signals. A FRI-based sparse observation model is constructed to address this issue, with the derivation as follows.

To overcome the limitation of the fixed window in the basic STFT, this paper adopts a causal analysis window associated with Infinite Impulse Response (IIR) filters to replace the traditional fixed window [19]. The window function is defined as:

*w_λ_*_,*α*_(*n*) = *γ^n^u*(*n*), *n* ≥ 0 (4)

where *γ* controls the attenuation speed of the window. The closer *γ* is to 1, the larger the effective width of the window (resulting in high frequency resolution); the closer *γ* is to 0, the smaller the effective width of the window (resulting in high time resolution). *u*(*n*) is the unit step function, where *u*(*n*) = 1 for *n* ≥ 0, and *u*(*n*) = 0 otherwise. This ensures the causality of the window function, i.e., only the current and past signal information is utilized.

Using the redefined window function mentioned above, combined with the Fourier transform properties of exponential functions and the integration-by-parts rule, the improved window function-based STFT is derived as follows:

(5) $${\mathrm{ISTFT}}_{x}\left(n,m\right)=\sum_{l=-\infty }^{+\infty }s\left(l\right)w_{\lambda ,\alpha }\left(n-l\right)e^{-\mathrm{jml}}$$

IF is a core parameter for bearing fault feature extraction. Based on the characteristic that the time derivative of the phase of an AM-FM signal equals the instantaneous frequency, a time-domain partial derivative operation is performed on the Improved Short-Time Fourier Transform (ISTFT) time–frequency coefficients. The real part is extracted to mitigate the influence of noise and improve the robustness of IF estimation, yielding an accurate estimated value of the instantaneous frequency, which is given by the following formula:

(6) f^kn,m=Rej∂∂nISTFTxn,m·ISTFTxn,m−1

where ${\hat{f}}_{k}\left(n,m\right)$ is the estimated instantaneous frequency of the *k*-th signal component at the time–frequency point (*n*,*m*); Re{·} is the operator that extracts the real part of a complex number. Although ISTFT improves the time–frequency aggregation, the problem of time–frequency ridge divergence still exists under actual operating conditions, i.e., energy is dispersed around the true instantaneous frequency [20]. To address this issue, a SST post-processing operator is introduced to compress and aggregate the time–frequency energy of ISTFT, gathering the dispersed energy along the true instantaneous frequency ridge to further improve the resolution and readability of the time–frequency plane, which is given by the following formula:

(7) $$\mathrm{SST}\left(n,\xi \right)=\int_{\Omega }{\mathrm{ISTFT}}_{x}\left(n,m\right)\delta \left(\xi -{\hat{f}}_{k}\left(n,m\right)\right)\mathrm{dm}$$

where *SST*(*n*,*ξ*) is the time–frequency transform coefficient obtained by SST; *ξ* is the compressed frequency axis; *Ω* is the effective frequency range of the signal, covering the entire interval of the fault characteristic frequencies of wind turbine bearings; δ(·) is the Dirac delta function, which is non-zero only when its independent variable is 0, achieving accurate aggregation of the time–frequency energy.

The fault signals of wind turbine bearings exhibit strong aliasing characteristics, and the mode separation capability of SST for aliased components is limited. Therefore, a SET operation is introduced on the basis of SST to enhance modal separation effect [21]. First, frequency-domain normalization is performed on the SST results, and the modal weight is calculated to highlight the high-energy fault feature modes while suppressing low-energy interference and noise modes. The formula for the modal weight is as follows:

(8) $$\alpha_{k}\left(n,m\right)=\frac{{\left|{\mathrm{SST}}_{k}\left(n,m\right)\right|}^{2}}{\sum_{m\in \Omega }{\left|{\mathrm{SST}}_{k}\left(n,m\right)\right|}^{2}}$$

where *α_k_*(*n*,*m*) is the modal weight of the *k*-th signal component at the time–frequency point (*n*,*m*), with a value range of [0, 1]. A larger value indicates a higher probability that the time–frequency point belongs to the fault feature mode. *SST_k_*(*n*,*m*) is the SST time–frequency coefficient of the *k*-th signal component.

The modal weight, time–frequency coefficients of ISTFT and estimated instantaneous frequency are fused. Weighted energy compression is realized by the Dirac delta function, achieving the high-resolution time–frequency representation of strongly modulated signals. The final expression of the time–frequency coefficients is obtained as:

(9) $$\mathrm{SET}\left(n,\xi \right)=\sum_{m}{\mathrm{ISTFT}}_{x}\left(n,m\right)\alpha_{k}\left(n,m\right)\delta \left(\xi -{\hat{f}}_{k}\left(n\right)\right)$$

### 2.2. Determination of Adaptive Window Width

To further optimize the above ISTFT framework proposed in Section 2.1, local Rényi entropy is introduced as a quantitative metric for time–frequency aggregation, so that window function parameters can be dynamically adjusted according to the local time-varying characteristics of the signal and realize adaptive time–frequency resolution optimization [22]. The local Rényi entropy, defined based on the TFA results, is expressed as follows:

(10) $$E_{R}\left(n,\lambda \right)=-\sum_{l\in N\left(n\right)}\sum_{m}{\left|P_{\lambda }\left(l,m\right)\right|}^{2\beta }\log\left({\left|P_{\lambda }\left(l,m\right)\right|}^{2\beta }\right)$$

(11) $$P_{\lambda }\left(l,m\right)={\left|{\mathrm{ISTFT}}_{x}\left(l,m;\lambda \right)\right|}^{2}$$

where *E_R_*(*n*,*λ*) is the local Rényi entropy at time *n* when the window width parameter is *λ*; N(n) is the time neighborhood, taking 5~10 sampling points around time *n* to ensure locality of the evaluation; *P_λ_*(*l*,*m*) is the time–frequency energy spectrum obtained by taking the modulus squared of the ISTFT time–frequency coefficients when the window width is *λ*, whose definition is consistent with that of the traditional STFT energy spectrum; *β* is the entropy index, which is set to 0.5 to balance the sensitivity and stability of the aggregation evaluation.

The optimal window width at each time point is solved by minimizing local Rényi entropy within sampling-rate-determined bounds, to achieve dynamic adaptation to local signal characteristics.

(12) $$\hat{\lambda }\left(n\right)=\underset{\lambda \in \left[\lambda_{\min},\lambda_{\max}\right]}{\mathrm{argmin}}E_{R}\left(n,\lambda \right)$$

where $\hat{\lambda }\left(n\right)$ is the optimal time-varying window width at time *n*; *λ_min_* and *λ_max_* are the lower and upper bounds of the window width, respectively, determined by the signal sampling rate to avoid meaningless values of the window width; argmin is the operator for finding the independent variable that minimizes the function. A low-pass filter is applied to the optimal window width sequence to suppress abrupt changes and obtain continuous time-varying parameters [23]. Compared with existing entropy-based adaptive time–frequency transforms that adopt fixed neighborhood radius or empirical parameter initialization, the presented method realizes fully data-driven window optimization. Narrow windows are selected for fast-varying impact features to achieve high time resolution, while wide windows are adopted for slowly varying harmonics to guarantee frequency accuracy, yielding more accurate time–frequency ridge characterization and better physical interpretability.

### 2.3. Proposed ISTFT-ASST

Based on the adaptive window optimization scheme derived in Section 2.2, the adaptive time-varying window width parameters are substituted into the improved STFT to obtain the time–frequency coefficients of ISTFT with adaptive window width. Then, the previously calculated modal weight and the estimated instantaneous frequency are fused to accurately gather the time–frequency energy along the true fault feature ridge. Finally, the core algorithm of the proposed ISTFT-ASST is derived. The final ISTFT-ASST time–frequency coefficients are defined in Equation (13), which integrates sparse preprocessing, adaptive windowing and modal weighting.

(13) $$\mathrm{ISTFT}\text{-}\mathrm{ASST}\left(n,\xi \right)=\sum_{m}{\mathrm{ISTFT}}_{x}\left(n,m;\hat{\lambda }\left(n\right)\right)\alpha_{k}\left(n,m;\hat{\lambda }\left(n\right)\right)\delta \left(\xi -{\hat{f}}_{k}\left(n,m;\hat{\lambda }\left(n\right)\right)\right)$$

where *ISTFT-ASST*(*n*,*ξ*) is the time–frequency transform coefficient of ISTFT-ASST for the *k*-th component; ${\mathrm{ISTFT}}_{x}\left(n,m;\hat{\lambda }\left(n\right)\right)$ is the ISTFT time–frequency coefficient corresponding to the adaptive time-varying window width $\hat{\lambda }\left(n\right)$; $\alpha_{k}\left(n,m;\hat{\lambda }\left(n\right)\right)$ is the modal weight of the *k*-th component calculated based on the adaptive window width, which is used to highlight the fault feature modes; ${\hat{f}}_{k}\left(n,m;\hat{\lambda }\left(n\right)\right)$ is the estimated instantaneous frequency of the *k*-th component calculated based on the adaptive window width.

The instantaneous amplitude is another core characteristic parameter for fault diagnosis of wind turbine bearings, and its amplitude is positively correlated with the fault severity [24]. Based on the time–frequency analysis results of ISTFT-ASST, the IA is accurately estimated by summing the absolute values of the time–frequency coefficients in the neighborhood of the instantaneous frequency of each fault component and taking the square root. The formula is as follows:

(14) $${\hat{A}}_{k}\left(n\right)=\sqrt{{\sum_{m\in \Omega_{k}}\left|{\mathrm{ISTFT}\text{-}\mathrm{ASST}}_{k}\left(n,m\right)\right|}^{2}}$$

where ${\hat{A}}_{k}\left(n\right)$ is the estimated IA of the *k*-th fault component at time *n*; *Ω_k_* is the neighborhood frequency range of the instantaneous frequency of the *k*-th fault component. Only the energy around the characteristic frequency is summed, which effectively avoids noise interference.

**Figure 1:**
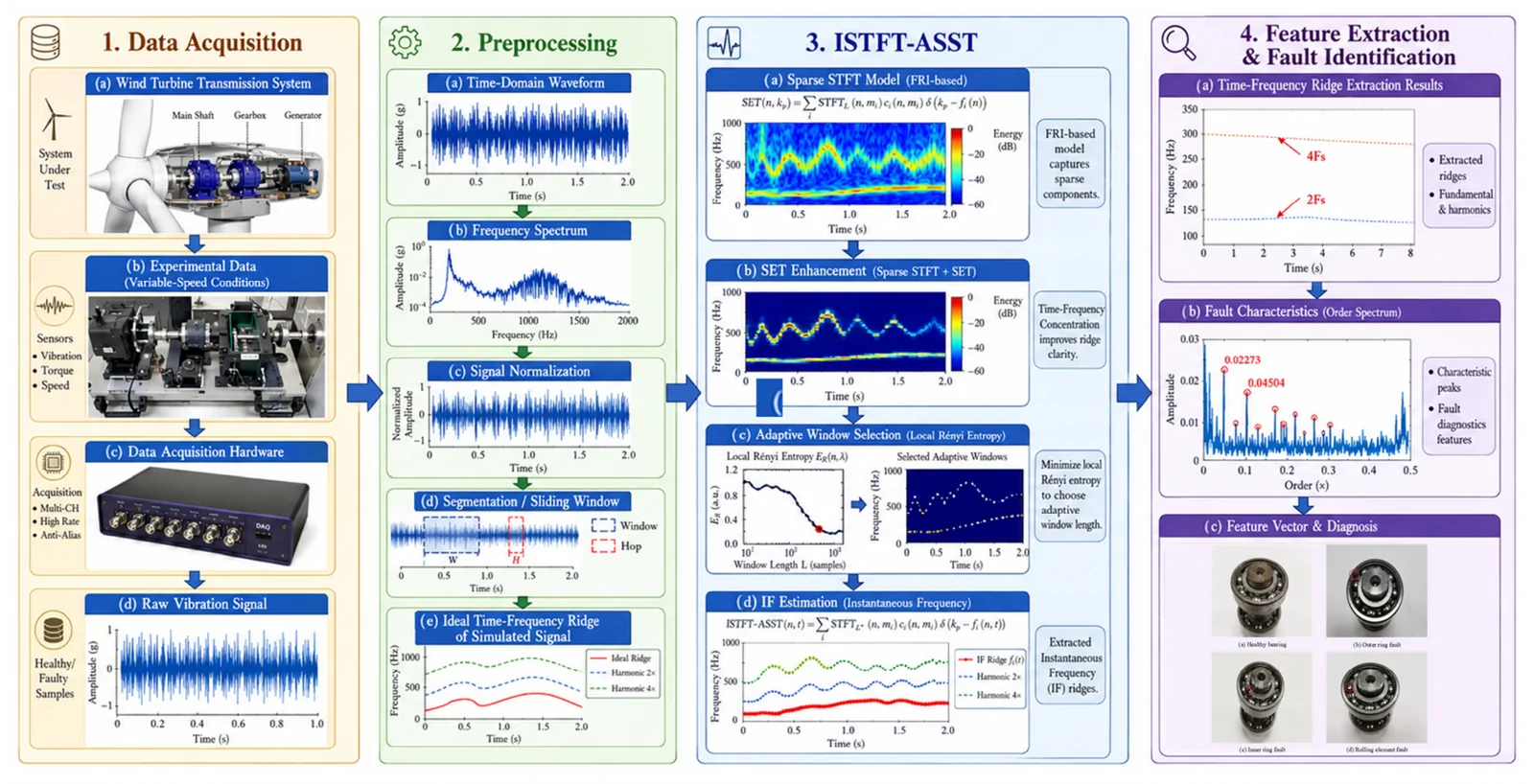
Overall research workflow of the proposed ISTFT-ASST method for wind turbine bearing fault diagnosis.

Different from conventional FRI-STFT that only pursues sparse representation, the proposed ISTFT-ASST combines FRI sparse observation modeling, SET modal weighting and local Rényi entropy-driven adaptive window optimization. The FRI sparse representation separates noise from valid fault-related components; SET modal weighting further suppresses interference time–frequency coefficients; entropy-guided adaptive window dynamically balances time–frequency resolution. The synergistic effect of the three modules enhances the decomposition capability for multi-component aliased signals under variable-speed conditions. The overall research workflow of the proposed ISTFT-ASST method is displayed in the following Figure 1.

## 3. Numerical Simulation Analysis

A multi-component simulation signal mimicking bearing outer-race faults was constructed to quantitatively evaluate the time–frequency resolution of ISTFT-ASST, with the mathematical expression as follows:

(15) $$\begin{array}{l} y_{1}=\sin(2\pi f_{1}(2{\left(t-1\right)}^{3}+6t^{2}))\\ y_{2}=\sin(2\pi (f_{2}t-4e^{-2t}(\sin(12\mathrm{\pi t}))))\\ s=y_{1}+y_{2}+y_{3} \end{array}$$

where *s* is a typical multi-component signal; *y*_1_ is a weakly frequency-modulated signal; *y*_2_ is a strongly frequency-modulated signal; and *y*_3_ is a Gaussian white noise signal with a noise standard deviation of 0.5. The signal sampling frequency is 2048 Hz, and the number of simulation points is 2048. The *f*_1_ = 10 Hz, and *f*_2_ = 300 Hz. The ideal instantaneous frequencies are *IF*_1_ = *f*_1_(6*t*^2^ + 6) and *IF*_2_ = *f*_2_ + 8*te*^−2*t*^sin(12*πt*) − 48*πe*^−2*t*^cos(12*πt*).

**Figure 2:**
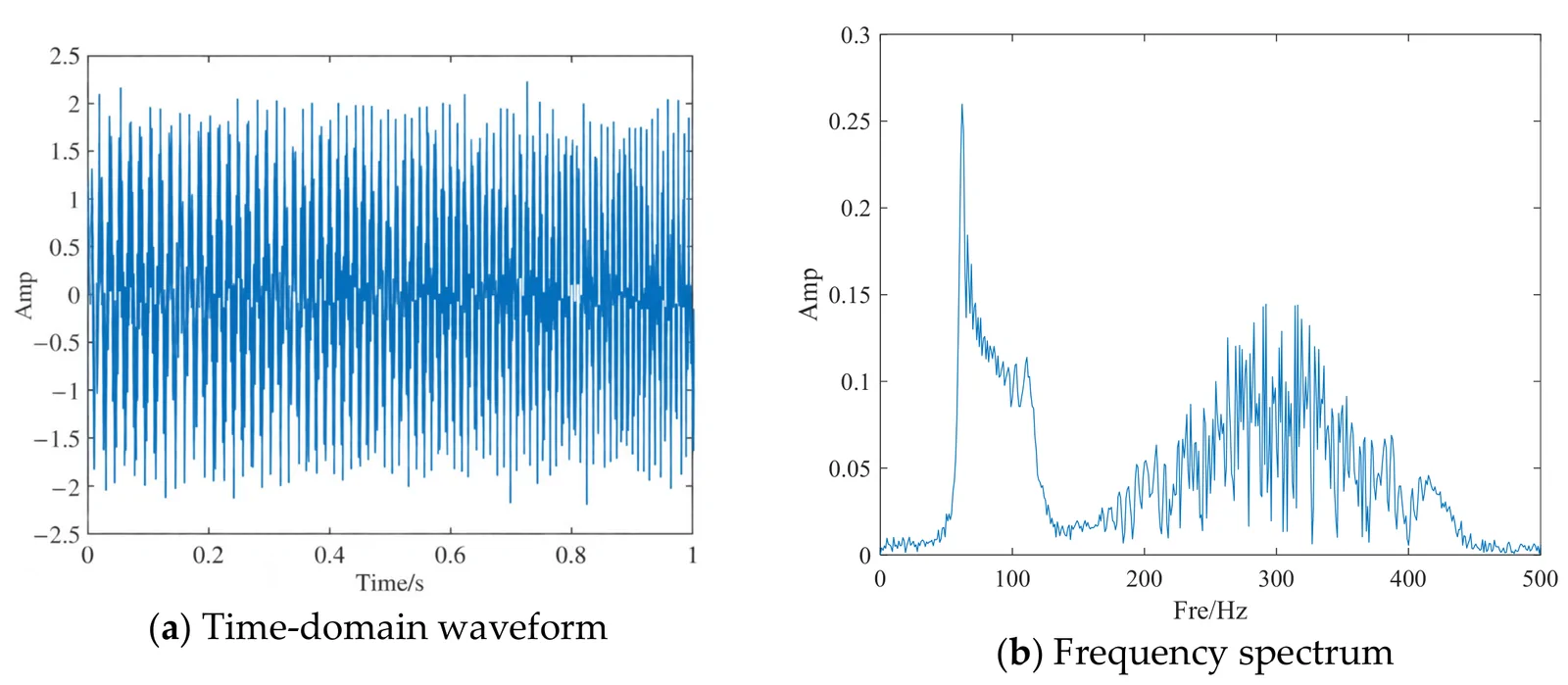
Time-domain waveform and frequency spectrum of the simulated multi-component non-stationary signal.

**Figure 3:**
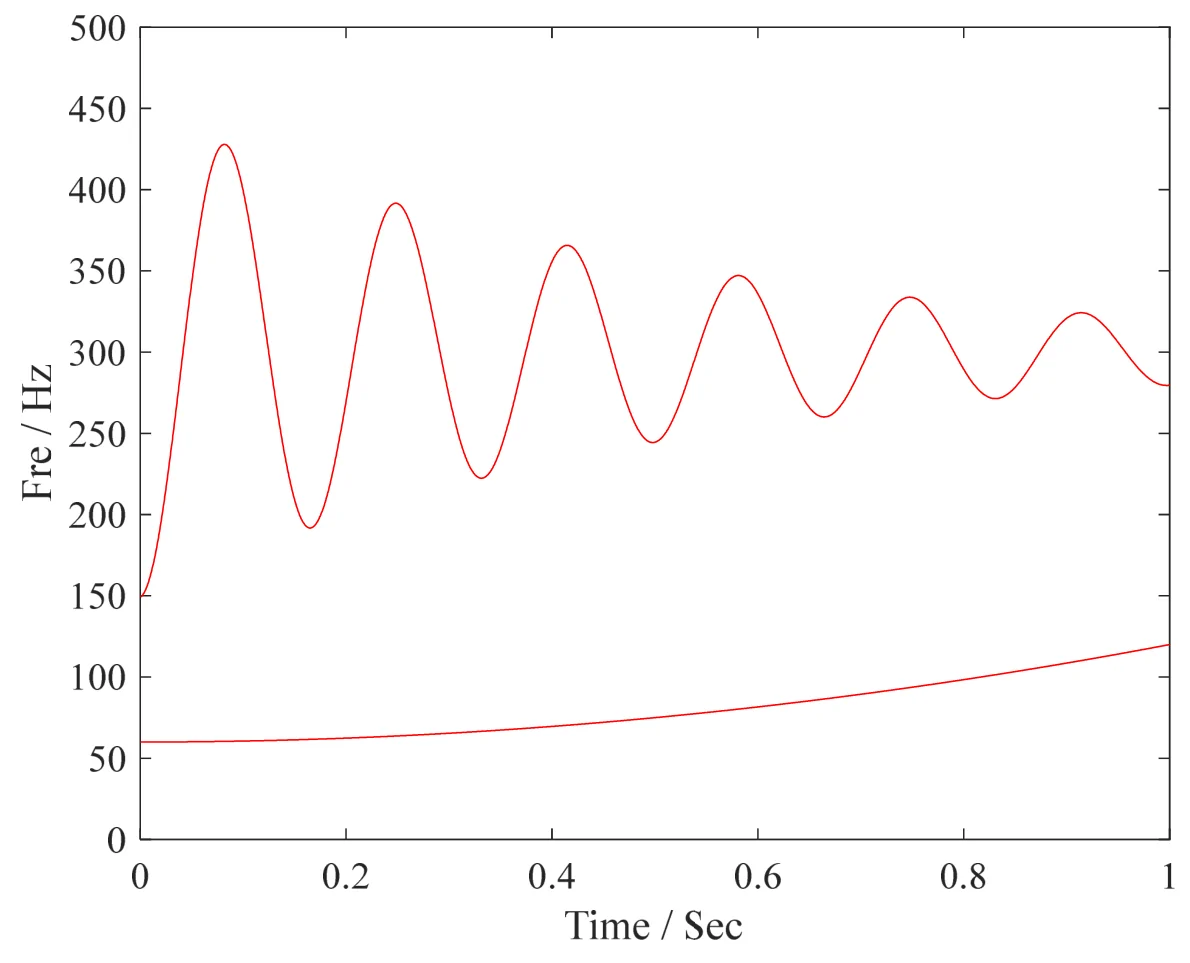
Ideal time–frequency ridge of the simulated signal.

The time-domain waveform and frequency spectra of the numerically simulated signal are shown in Figure 2. Due to its time-varying characteristics and noise interference, effective feature information can hardly be identified from either the time-domain waveform or the frequency spectrum [25]. The ideal IF representation of the simulated signal is shown in Figure 3. In general, the closer the processing output of the investigated time–frequency analysis method approximates the ideal IF curve, the better the time–frequency representation performance of the method [26].

**Figure 4:**
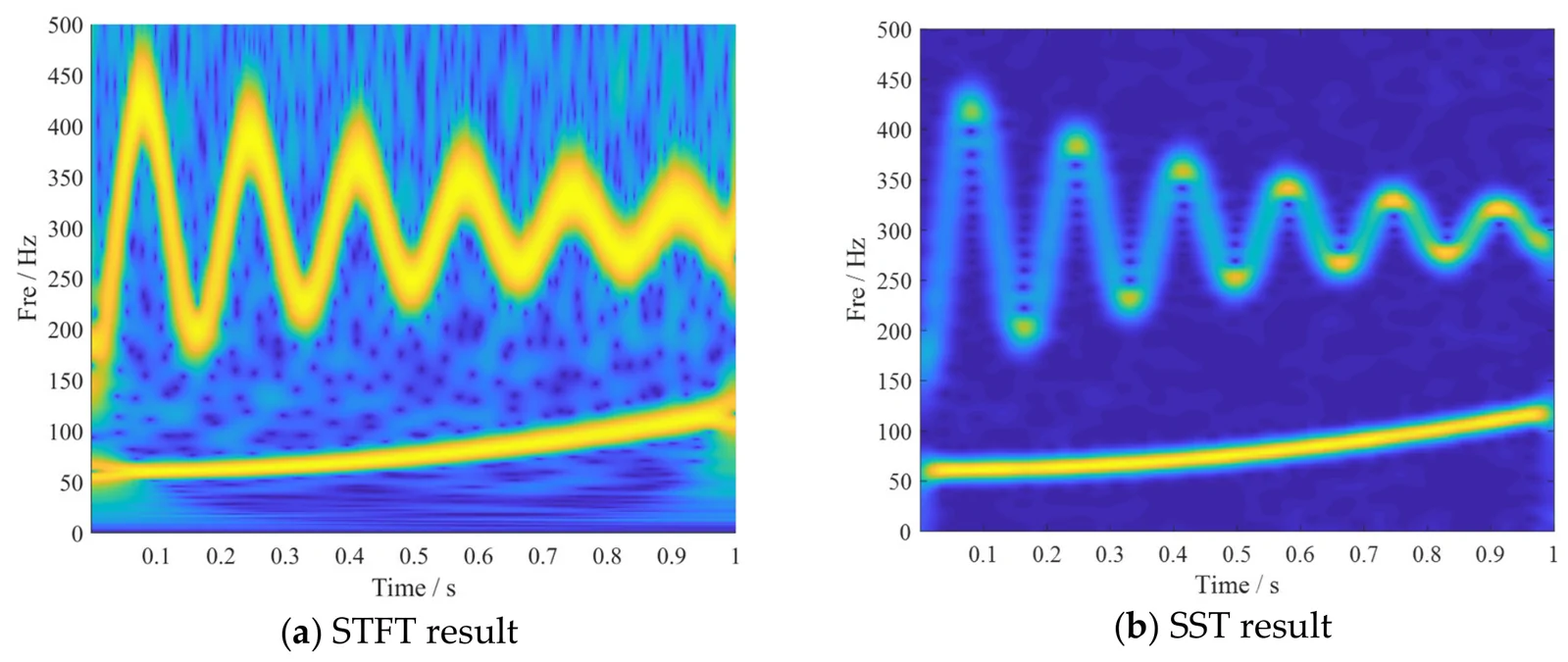
Time–frequency representations obtained by STFT and SST for simulated signal.

**Figure 5:**
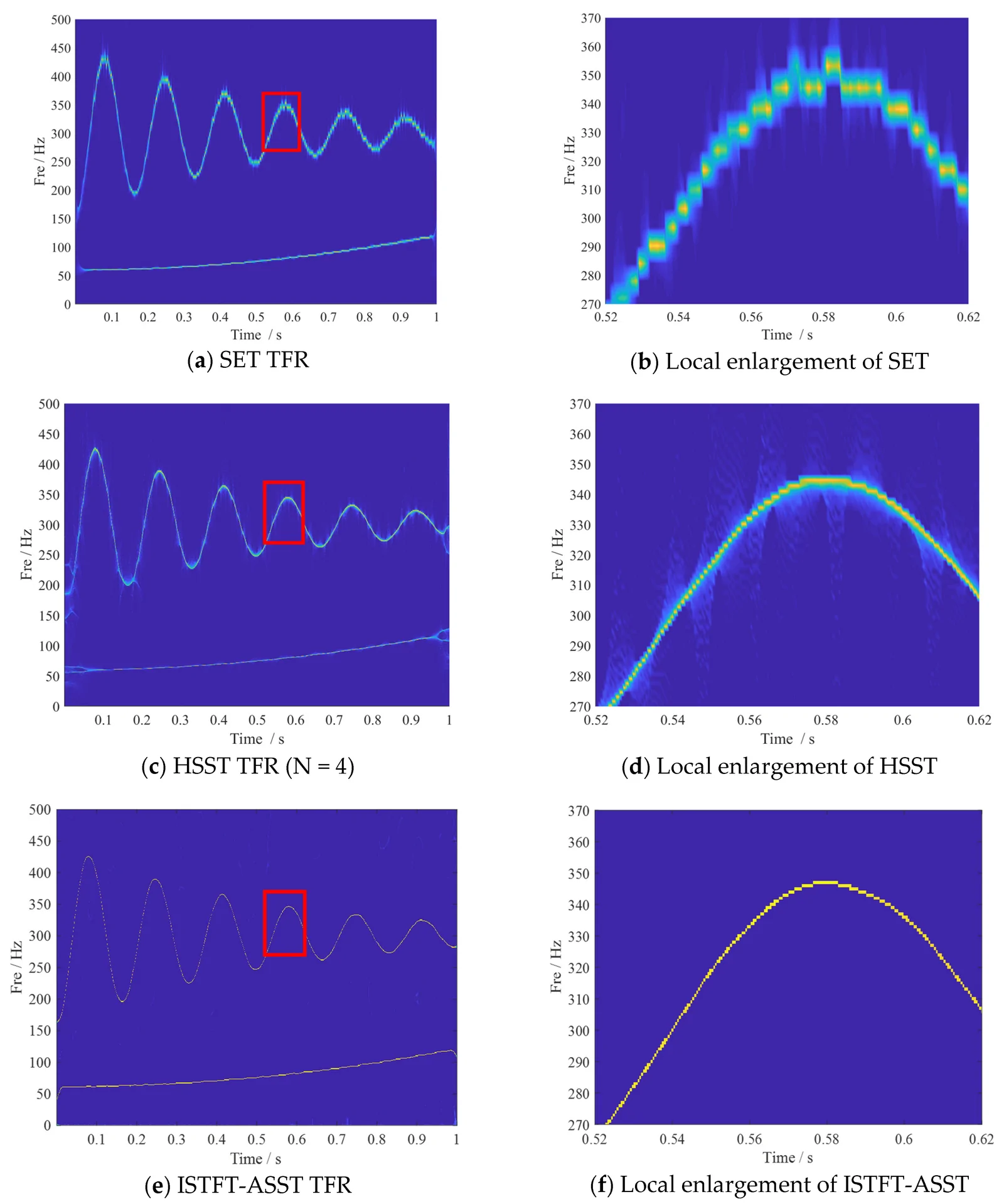
Time–frequency analysis results of SET, HSST and the proposed ISTFT-ASST on simulated signal, including local enlarged views of time–frequency ridges.

Considering that the fault signals of mechanical equipment often contain impulsive characteristics, the STFT usually adopts the Hanning window, with an overlap ratio of 50%. For the numerical simulation signal, the window width of the STFT was set to 100. For mechanical equipment vibration signals (such as gear meshing frequencies, bearing fault characteristic frequencies, etc.), their phase changes can usually be well approximated by low-order polynomials. A 4th-order Taylor expansion is already capable of capturing the majority of nonlinear frequency modulation characteristics in engineering scenarios. Therefore, the order of HSST was set at *N* = 4. The resulting time–frequency representations are presented in Figure 4 and Figure 5, and the quantitative comparison of energy concentration via Rényi entropy is summarized in Table 1.

Figure 4 presents the results of STFT and SST, both of which suffer from blurred time–frequency ridges and energy dispersion under fixed-window constraints. As shown in Figure 5, SET and HSST achieved improved aggregation to some extent, but still retained noticeable noise interference and ridge diffusion. In contrast, the proposed ISTFT-ASST (Figure 5e,f) achieved the sharpest time–frequency ridges. Compared with fixed-window STFT, more continuous time–frequency ridges obtained by the proposed method verify the effectiveness of such adaptive window adjustment.

Local Rényi entropy was employed as the quantitative metric, and the comparison results of Rényi entropy values for various TFA methods dealing with multi-component noisy signals are presented in Table 1. As indicated in Table 1, the proposed ISTFT-ASST achieved the minimum Rényi entropy, revealing that the proposed method provides superior energy concentration and achieves the optimal overall analysis performance [27]. The minimum Rényi entropy achieved by ISTFT-ASST demonstrates its superior energy concentration and favorable noise suppression capability.

Table 2 shows the implementation of ablation for the simulated signal. From Table 2, it can be observed that different modules still exhibit significant differences in terms of enhancing the time–frequency resolution and improving the reconstruction ability of useful signals. Based on the calculation results in Table 2, we can reasonably conclude that the time–frequency analysis method proposed in this paper, by integrating the advantages of the three modules, possesses a stronger time–frequency analysis capability.

**Figure 6:**
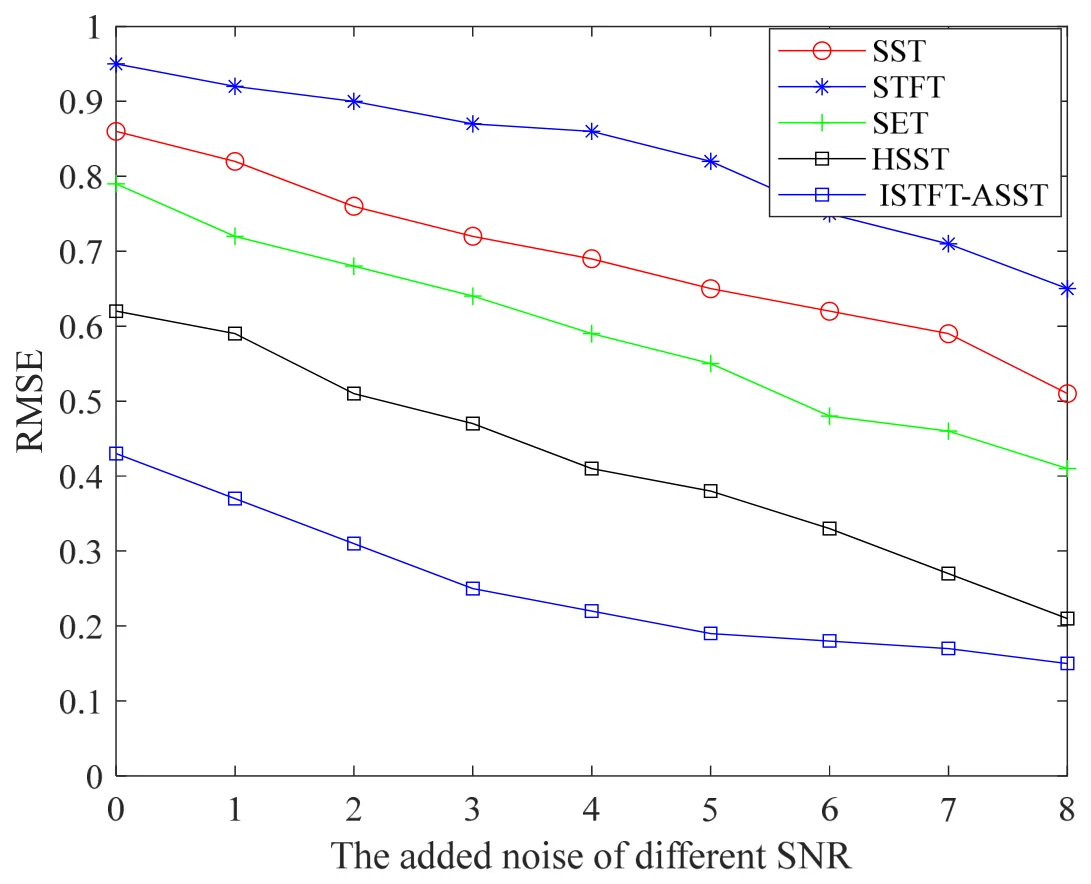
Comparison of RMSE at different signal-to-noise ratios by five methods.

Furthermore, to demonstrate the performance of the proposed ISTFT-ASST in signal reconstruction, the root mean square error (RMSE) of five methods was calculated for different signal-to-noise ratios (SNRs) for the simulation signals. Figure 6 displays the variation curves of the RMSE after calculating through the five methods: STFT, SST, SET, HSST, and the proposed ISTFT-ASST. It displays the RMSE between the noisy simulation signal and the reconstructed results of different methods. The smaller the RMSE, the better the signal reconstruction effect. Clearly, the method proposed in this paper can achieve high-resolution time–frequency representation of complex multi-component signals. Additionally, by observing the RMSE variation curves of the five methods, it was found that the RMSE between ISTFT-ASST and the original signal was the smallest, indicating that the proposed method can effectively restore the original signal even in the presence of strong noise.

## 4. Experimental Analyses

### 4.1. Parameter Configuration

For the conventional comparison methods (STFT, SST, SET, and HSST), the parameters are set according to well-established empirical rules. Since the Synchrosqueezing transform essentially reassigns the time–frequency coefficients of the STFT, the window function and the window length play a central role in these methods. For mechanical vibration signals with impulsive characteristics, the Hanning window is commonly adopted to suppress spectral leakage. In principle, when the signal contains stationary or slowly-varying components, the window length may be set to 2–4 times the number of sampling points corresponding to the longest characteristic frequency period. The overlap ratio is set to 50% as a trade-off between temporal resolution and computational cost. Regarding the IF estimation, the phase of the gear meshing and bearing fault vibration components can usually be well approximated by low-order polynomials. A fourth-order Taylor expansion is adopted because it captures the dominant nonlinear frequency modulation while avoiding the limited accuracy gain and the extra computational burden of higher-order expansions. For the HSST, the redistribution order *N* = 4 is used, following the recommendation in the original literature to balance the energy concentration and the computational overhead.

In contrast, the proposed ISTFT-ASST adopts a fully adaptive parameter optimization strategy. Most core time–frequency parameters are determined during the computation rather than preset manually. Specifically, the time-varying window width is dynamically optimized by minimizing the Rényi entropy criterion, which automatically matches the time–frequency variation characteristics of the vibration signal under variable-speed conditions. Only a few basic acquisition parameters, such as the sampling frequency, the signal segment length, and the overlap ratio, are fixed according to the actual measurement environment, following the general practice in time–frequency analysis.

### 4.2. Fault Diagnosis of Bearing Faults Using a Laboratory Test Rig

**Figure 7:**
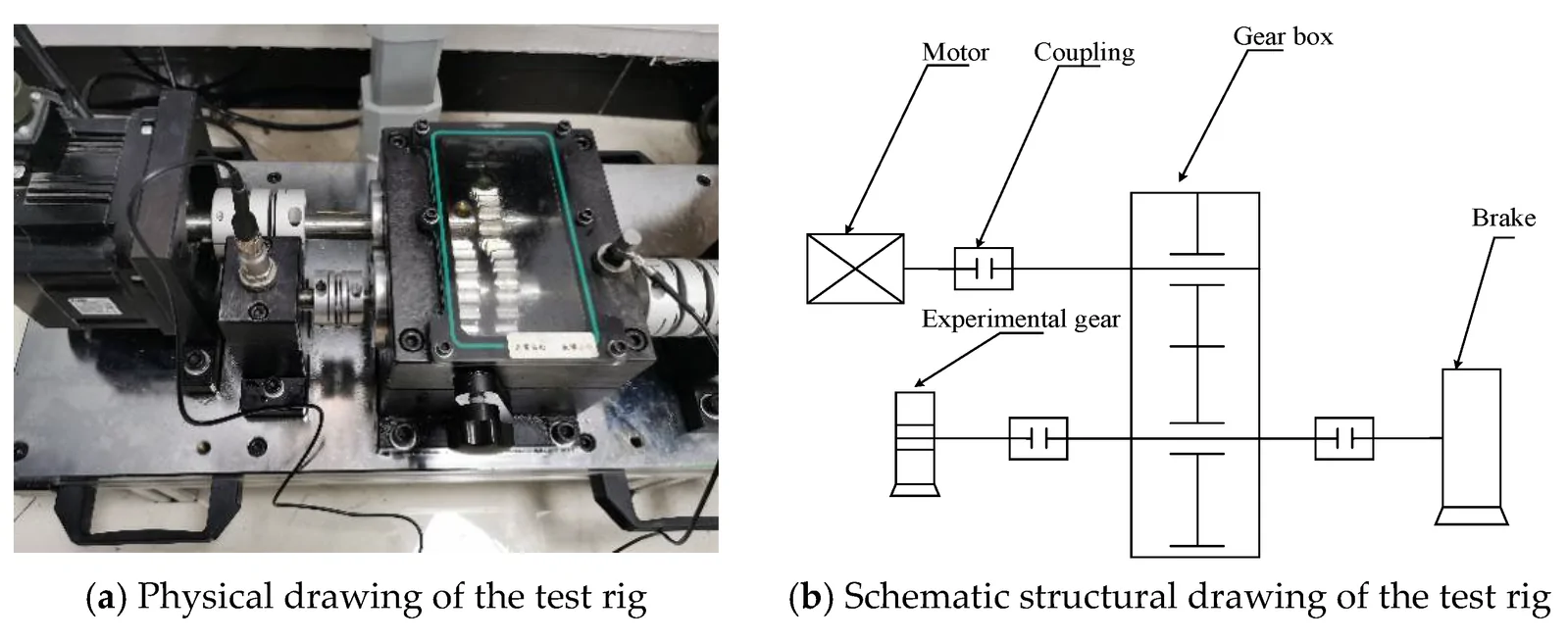
Physical photograph and structural schematic of the fault test bench.

**Figure 8:**
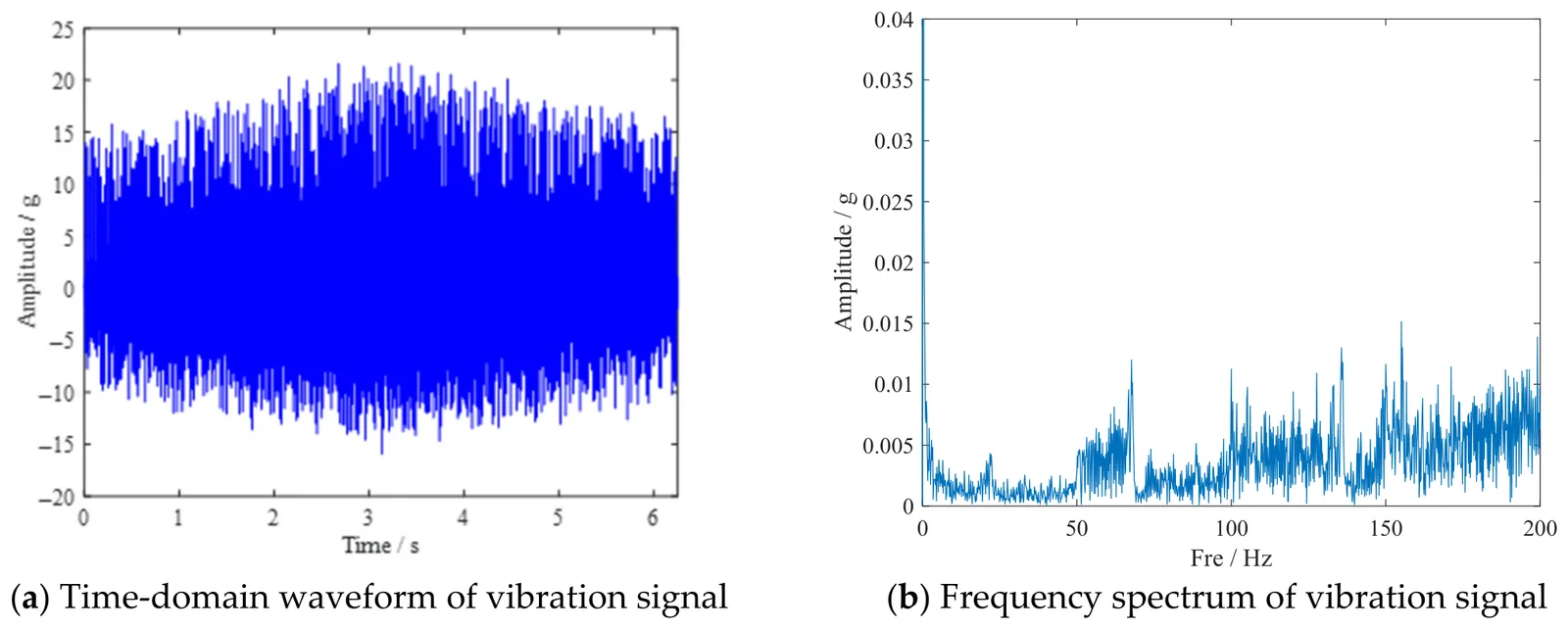
Time-domain waveform and frequency spectrum of variable-speed outer-race fault bearing vibration signal.

Variable-speed bearing fault data from a laboratory test rig were further used to verify the practical performance of ISTFT-ASST. The experimental data were obtained from a self-developed bearing-gear comprehensive fault test rig in our laboratory, as illustrated in Figure 7. Vibration signals were collected from rolling bearings (model 6202, Svenska Kullager-Fabriken, Sweden) with outer ring defects. The sampling frequency was set to 2560 Hz, with a total sampling duration of approximately 6 s. During the experiment, the rotational speed was accelerated from 0 r/min to 2000 r/min and then decelerated to 0 r/min. The shaft frequency ranged from 0 to 33.3 Hz. The outer race fault frequency (BPFO) was calculated from the bearing geometric parameters and the shaft rotational speed, where the shaft speed was derived from the motor speed through the gearbox reduction ratio. The resulting BPFO varied from 0 to 68 Hz. Figure 8a,b show the time-domain waveform and the frequency spectrum of the vibration signal. Because noise and other interference will affect the identification of bearing fault characteristic frequency, the outer ring fault frequency cannot be accurately obtained according to the time-domain and frequency-domain characteristics in the figure. Therefore, it is necessary to analyze the signal using dedicated TFA methods [28].

**Figure 9:**
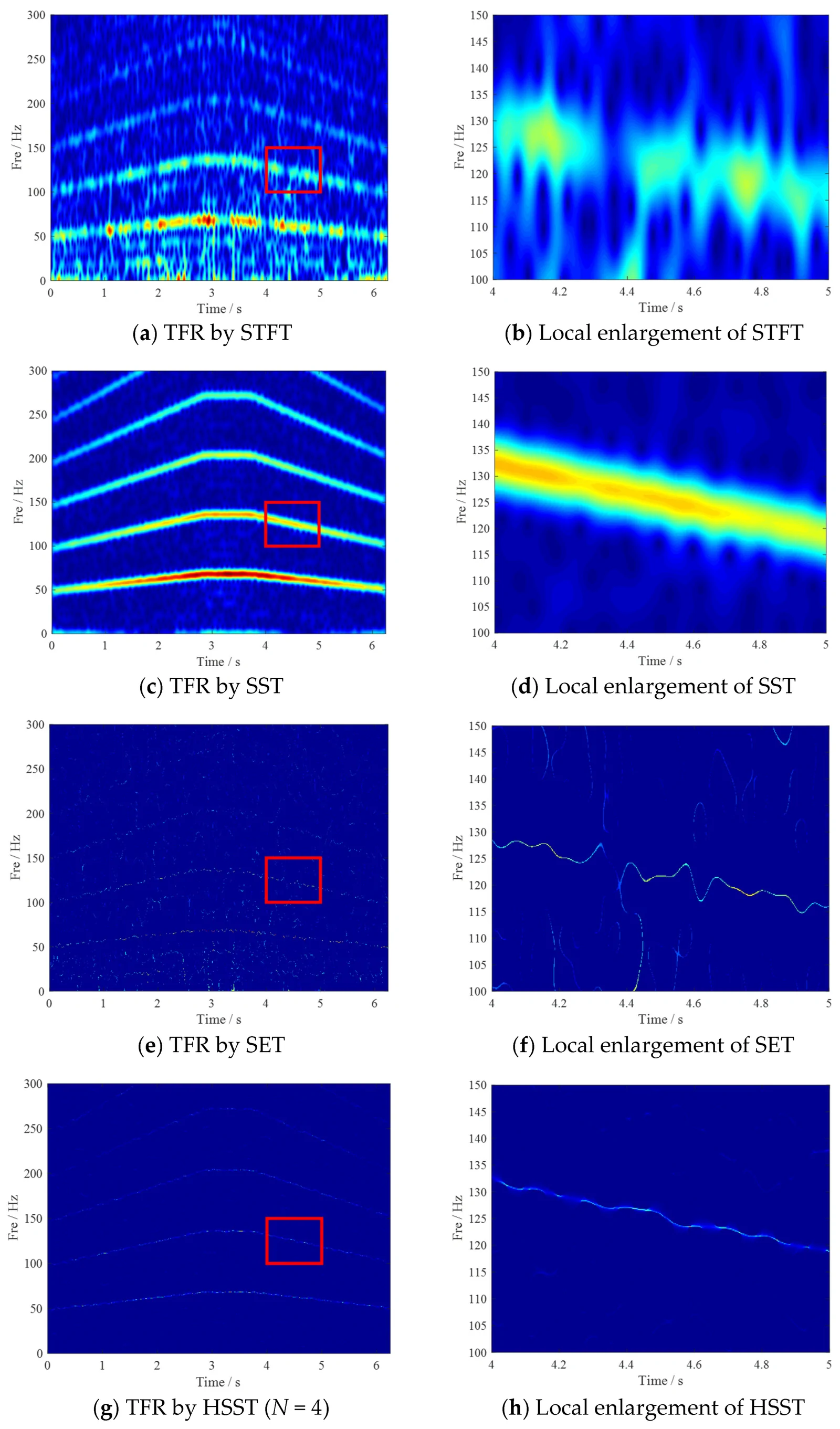
Time–frequency representations and local amplification effects of STFT, SST, SET and HSST for variable-speed bearing fault vibration signal.

In this experiment, the commonly used STFT, SST, SET and HSST (N = 4) were selected as comparative analysis methods to evaluate the fault feature extraction performance of the proposed ISTFT-ASST for bearing vibration signals under variable-speed conditions. The parameter configurations of these comparison methods are consistent with the optimal settings described in Section 4.1, ensuring a fair and reproducible comparison. Meanwhile, to make the fault characteristic frequencies more distinguishable, the local signal segment was extracted and analyzed. The time–frequency results calculated by the above four comparative methods are shown in Figure 9a–h, with the locally amplified effects on the right side of each figure. It can be observed from Figure 9b that the time–frequency features obtained by STFT exhibit significant blurring, accompanied by prominent energy dispersion in the vicinity of the time–frequency ridges. In comparison with SST, both SET and HSST (displayed in Figure 9f,h) achieved a certain degree of improvement in representing time-varying signals, with energy concentrated relatively well along the ridges. Nevertheless, the time–frequency ridges at high frequencies still exhibit blurring, and the low-resolution time–frequency distributions hinder the effective extraction of ridge features.

**Figure 10:**
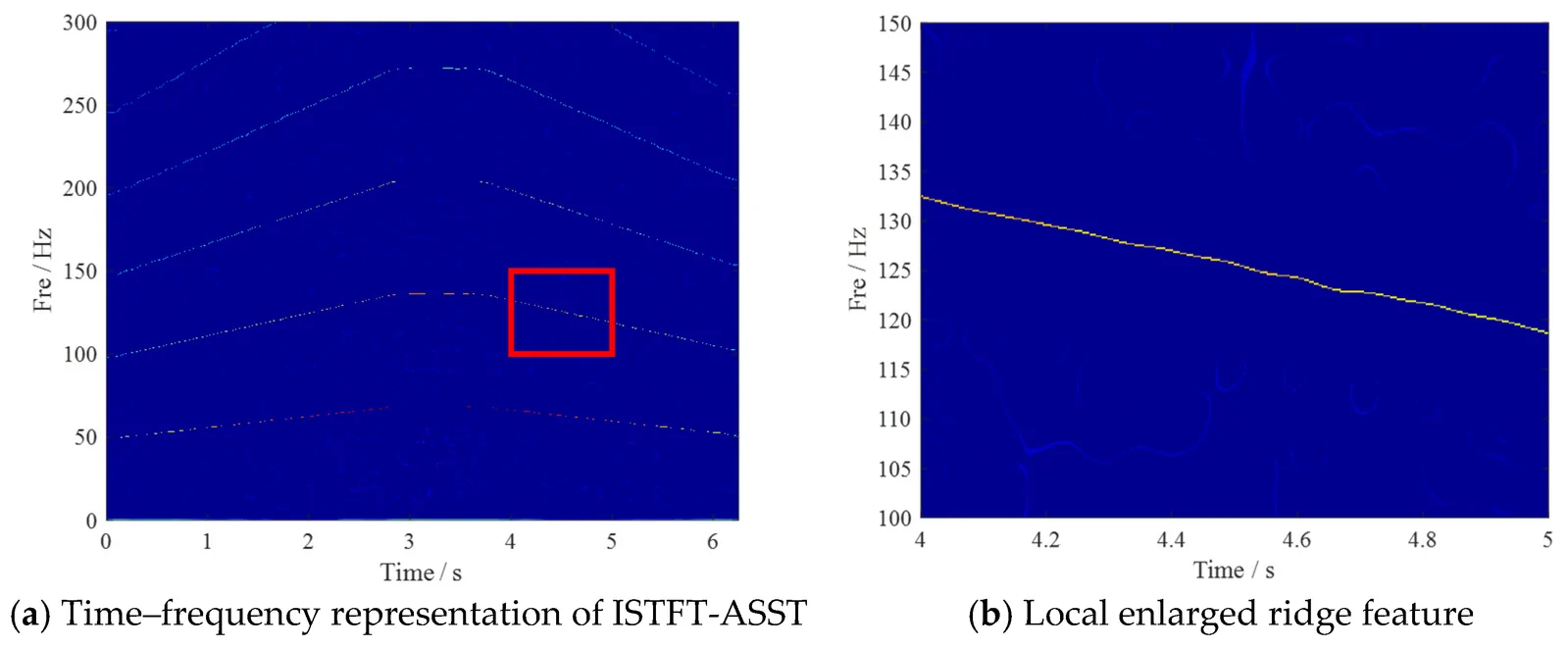
Time–frequency representation of bearing fault signal by the proposed ISTFT-ASST.

**Figure 11:**
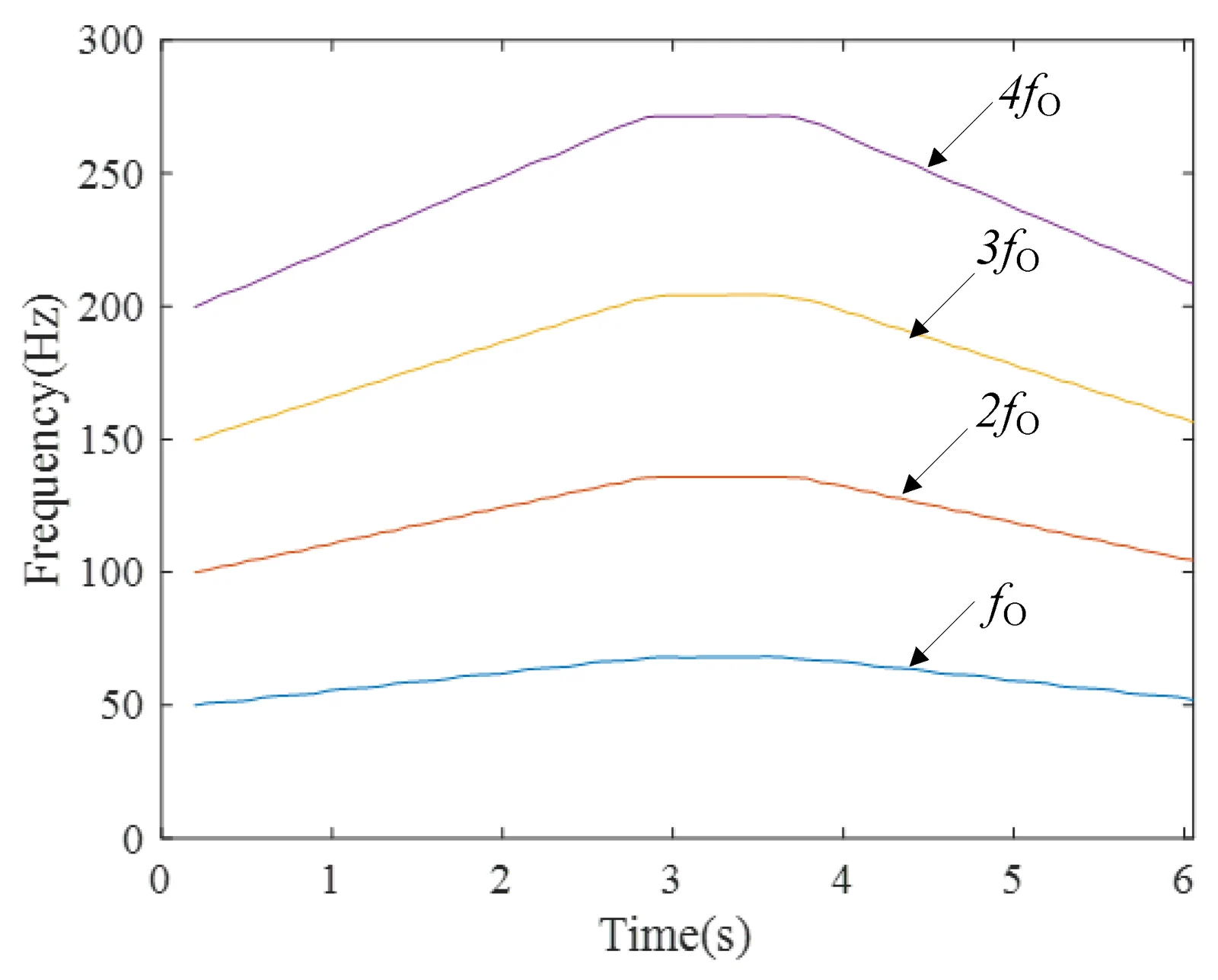
Instantaneous frequency ridge extraction results of bearing fault signal based on ISTFT-ASST.

Subsequently, the proposed ISTFT-ASST method was used to analyze the vibration signals of rolling bearings, and the corresponding time–frequency representation results are shown in Figure 10a,b. The ridge line feature extraction results of each time–frequency plane are then presented in Figure 11.

TFA ultimately aims to identify the correspondence between time–frequency ridges and time-varying characteristic frequency components [29]. It can be seen in Figure 9 that the results calculated by STFT, SST, SET and HSST (*N* = 4) all have different degrees of distortion. In contrast, Figure 10 and Figure 11 show that the presented ISTFT-ASST achieved a higher time–frequency resolution in identifying time-varying fault features and also exhibited excellent ridge line extraction performance. Finally, the Rényi entropy values calculated by the five time–frequency analysis methods were compared. The results are shown in Table 3.

**Figure 12:**
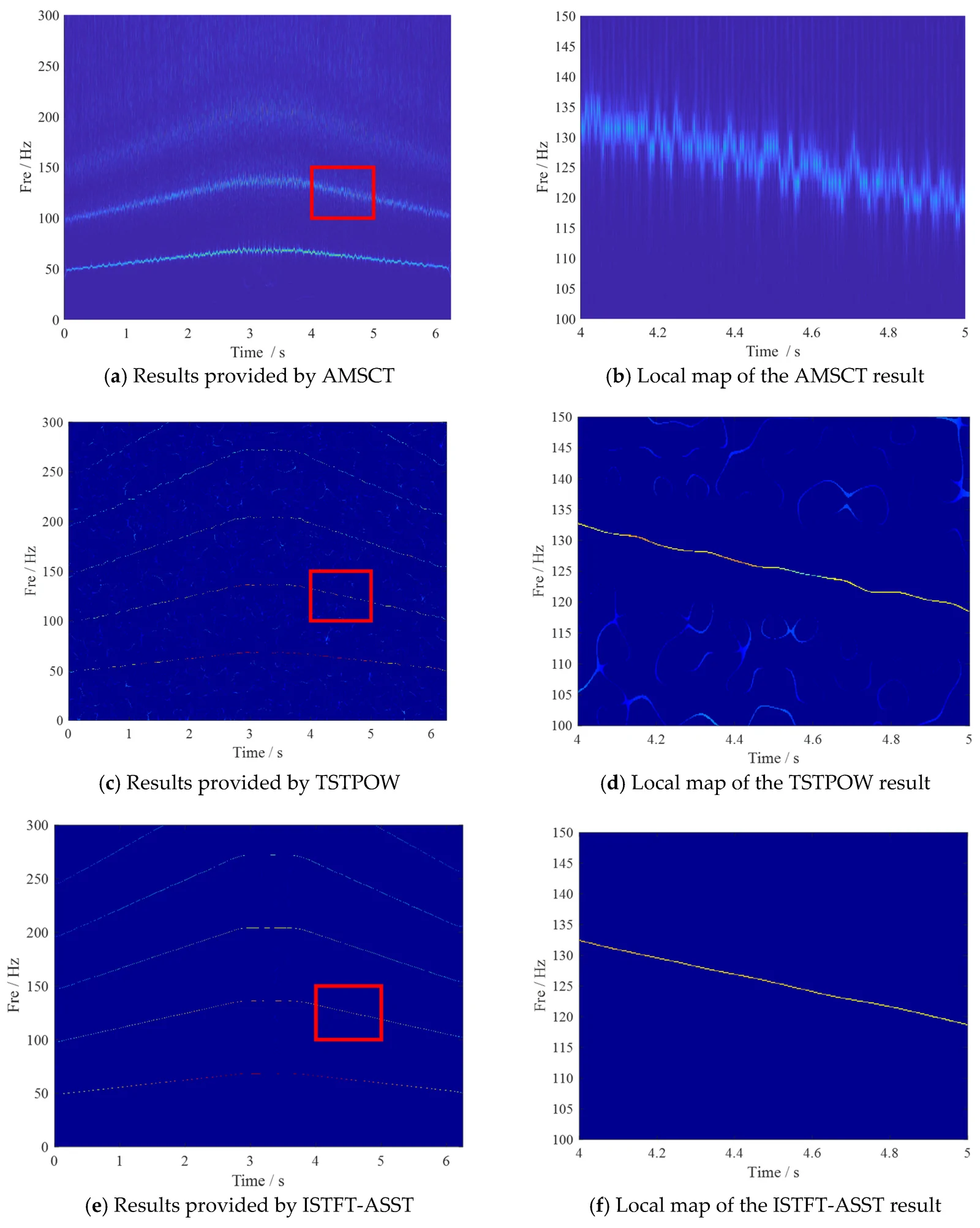
Time–frequency representations of several different methods.

To further demonstrate the competitiveness of the proposed method, we compared it with two state-of-the-art methods, AMSCT and TSTPOW, using the same variable-speed outer-race fault bearing data described above. Figure 12 displays the time–frequency representations obtained by AMSCT, TSTPOW and ISTFT-ASST. It can be seen that the proposed ISTFT-ASST yielded the clearest and most concentrated time–frequency plane. Table 4 lists the Rényi entropy values of the three methods. The lowest value achieved by ISTFT-ASST confirms its superior energy concentration.

### 4.3. Fault Diagnosis of Main Shaft Bearings in a Direct-Drive Wind Turbine

**Figure 13:**
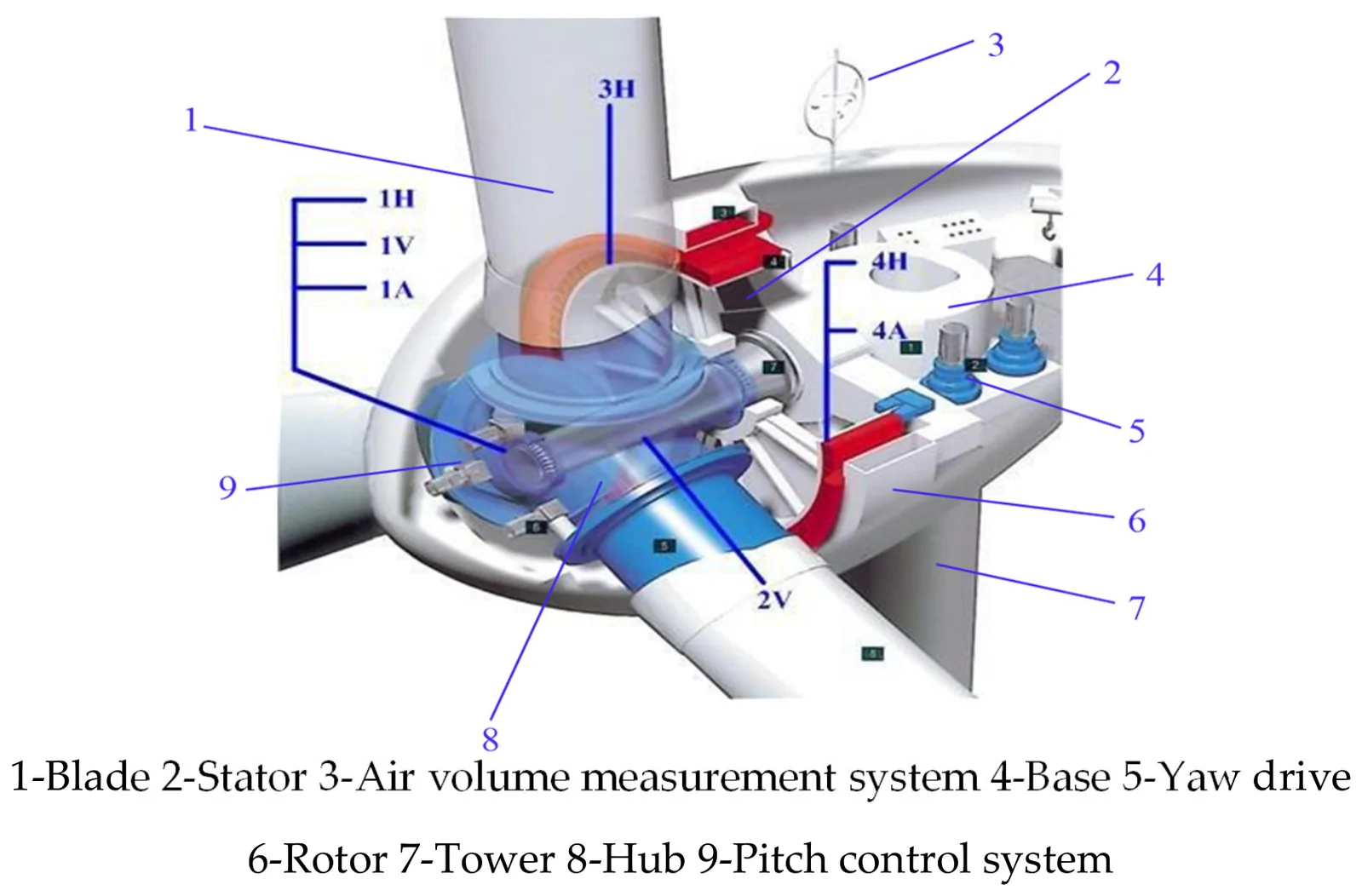
Structure of the direct-drive permanent magnet wind turbine and layout of sensor measurement points.

To verify the feasibility of the proposed method for analyzing the actual vibration data of wind turbines, vibration time–frequency analysis data were acquired from a 1.5-MW direct-drive permanent magnet wind turbine, which has a maximum rotational speed of 17.3 r/min, a rated power of 1580 kW, a rotational speed range of 9–17.3 r/min, and a total mass of 43.8 t. Rolling bearings play a crucial supporting role in wind turbines, serving as the supporting components for the main shaft, yaw system, pitch blades and the entire wind turbine system. In particular, the main shaft bearings are vulnerable and failure-prone components. The wind turbine’s main shaft bearings are divided into front and rear bearings, with the front bearing being a double-row tapered roller bearing, and the rear bearing being a single-row cylindrical roller bearing [30]. The layout of the wind turbine’s main components and sensor measurement points is presented in Figure 13, where 1H represents the horizontal direction of the main bearing, 1V the vertical direction, and 1A the axial direction.

**Figure 14:**
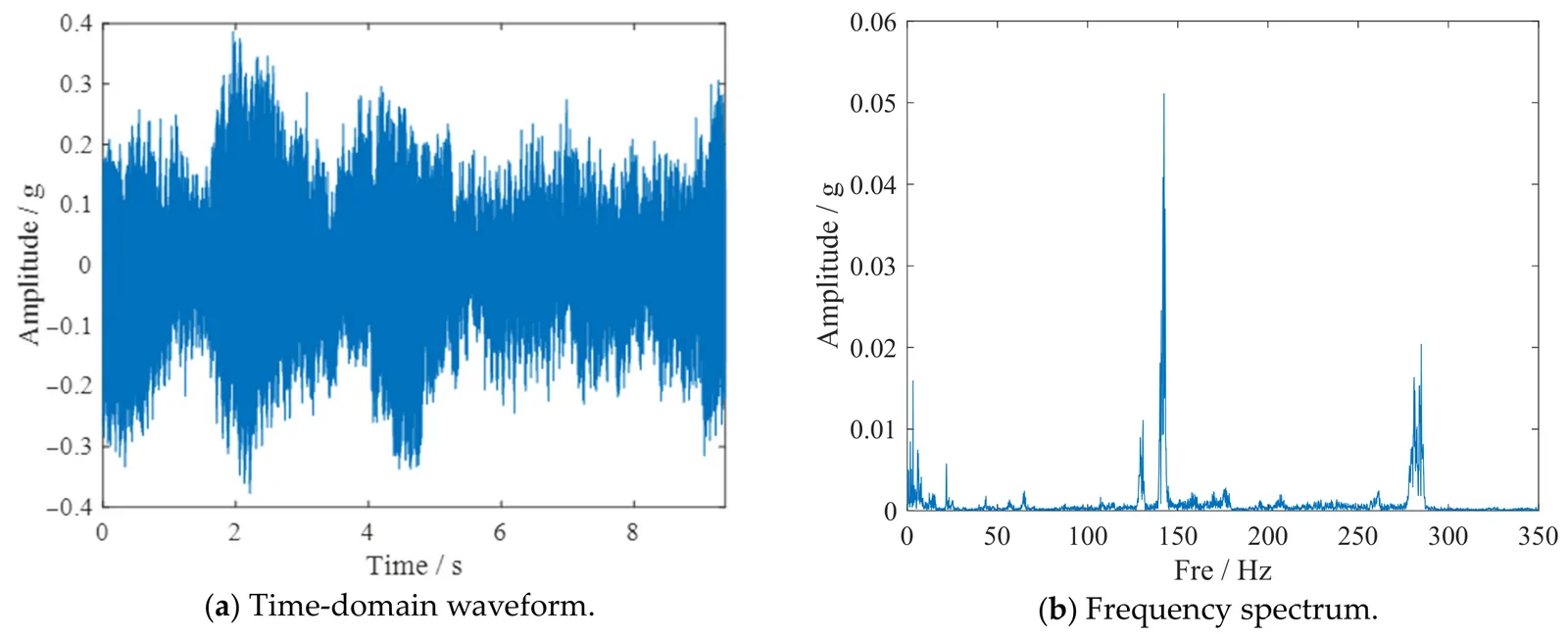
Time-domain waveform and frequency spectrum of measured vibration signal from wind turbine main bearing.

**Figure 15:**
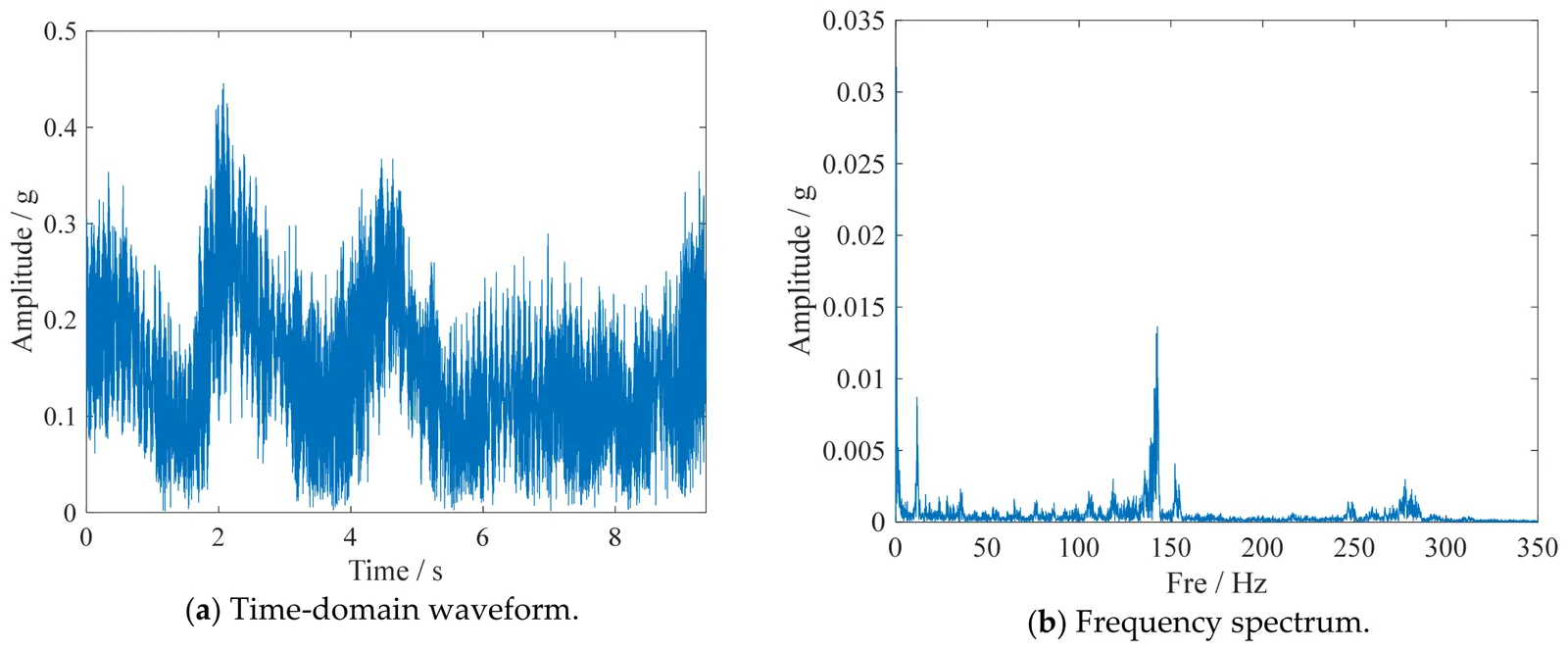
Time-domain waveform and envelope frequency spectrum of measured wind turbine vibration signal.

The proposed method ISTFT-ASST was applied to analyze the vibration signals of rolling bearings in the direct-drive wind turbine unit. The rotational speed of the wind turbine ranged from 9 r/min to 17.3 r/min, corresponding to a rotational frequency range of 0.1667 Hz to 0.2883 Hz. In addition, the slot passing frequency ranged from 48 Hz to 83 Hz, the double slot passing frequency from 96 Hz to 166 Hz, and thus the quadruple slot passing frequency (4F_s_) was calculated to range from 192 Hz to 332 Hz. Table 5 details the mechanical characteristic frequencies of the wind turbine, and Table 6 presents its electrical characteristic frequencies, where fr represents the time-varying rotational frequency. This paper mainly analyzes the vibration signals collected from the horizontal measurement point (1H) of the rolling bearing. The time-domain waveform and frequency spectrum of the vibration signal are shown in Figure 14, and the results of the envelope spectrum analysis are presented in Figure 15.

Under constant rotating speed conditions, the frequency spectral features of the double and quadruple slot passing frequencies for this turbine model are distinct and clear. In the event of a bearing fault, the fault characteristic frequencies and their harmonic components can also be identified via conventional envelope spectrum analysis. However, under variable rotating speed conditions, the double and quadruple slot passing frequencies in the frequency spectrum become distributed discretely [31]. For the measured fault data of the wind turbine, Figure 14b shows the results obtained by FFT and Figure 15b the results from envelope spectrum analysis, from which neither the fault characteristic frequency nor its harmonics can be identified. In addition, in the early stage of bearing fault development, the amplitude and fault characteristics are weak, making it impossible to distinguish and extract the characteristic frequency in the envelope spectrum.

**Figure 16:**
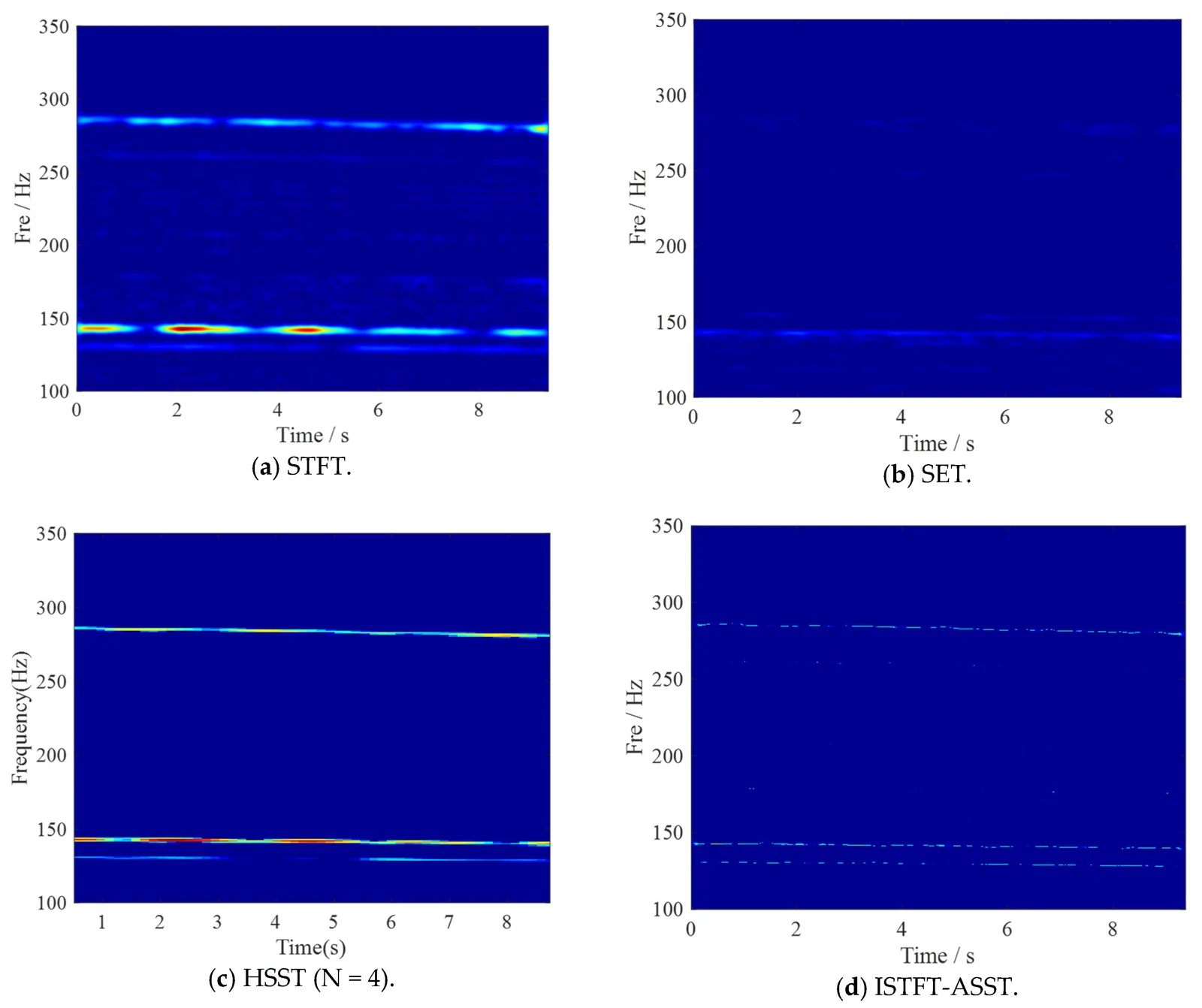
Time–frequency representations of wind turbine bearing vibration signal processed by STFT, SET, HSST (*N* = 4) and ISTFT-ASST.

**Figure 17:**
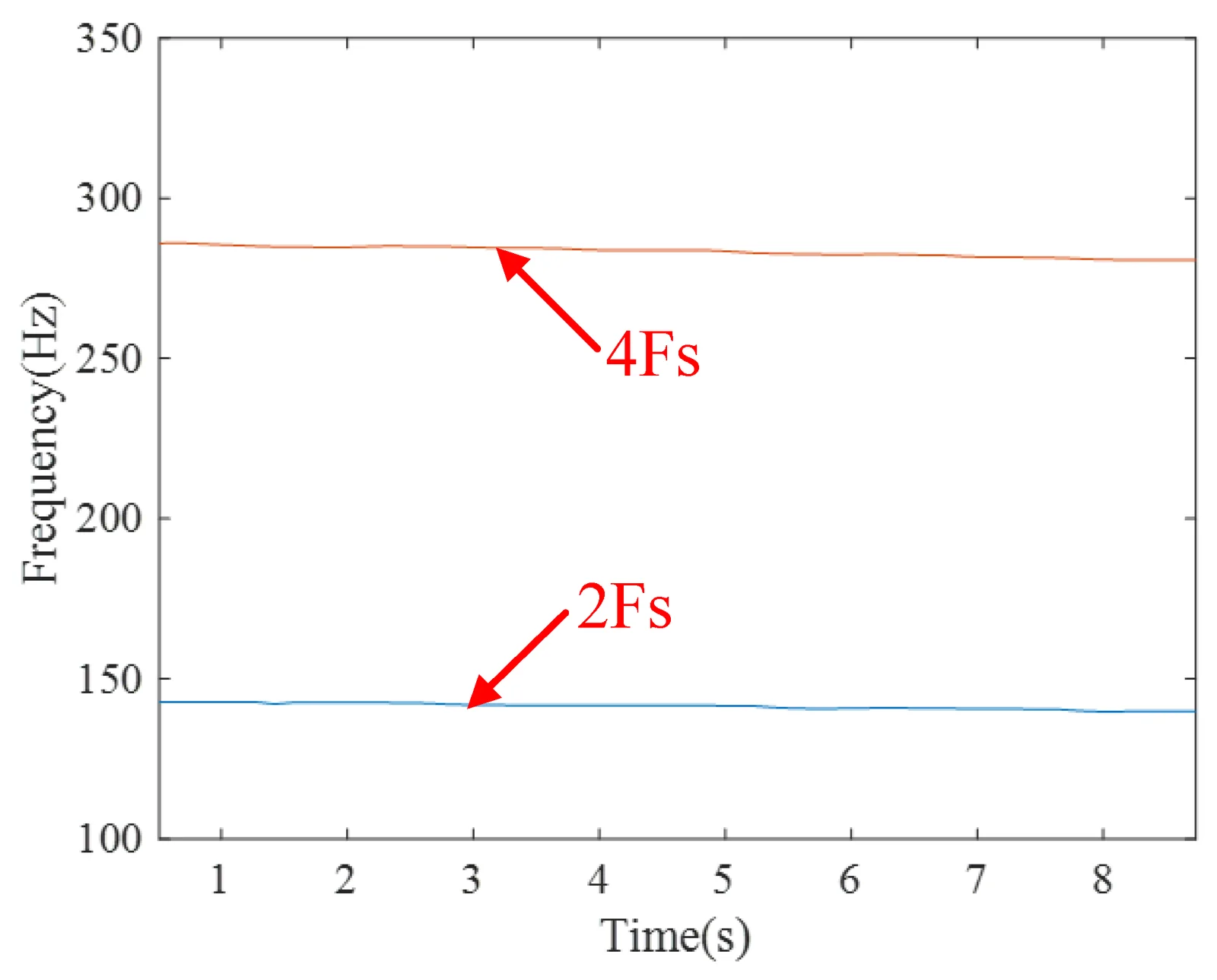
Time–frequency ridge extraction results of wind turbine bearing signal using ISTFT-ASST.

TFA is an important tool for fault diagnosis of wind turbines under variable rotational speed conditions [32]. Through an ideal time–frequency representation, time-varying fault features can be accurately located on the time–frequency plane. Based on the above methods, Figure 16 plots the time–frequency representation results generated by STFT, SET, HSST (*N* = 4) and the ISTFT-ASST method proposed in this paper. Since the wind speed changed slowly, the vertical coordinate range was limited to 100–350 Hz to more clearly identify the variation law of fault features with time. As STFT is suitable for weakly modulated and slowly varying signals [33], only a blurred time–frequency representation can be observed in Figure 16a, so its time–frequency resolution needs to be improved. SET only focuses on the time–frequency coefficients at the immediate position, and its time–frequency analysis results are shown in Figure 16b, where the time-varying double and quadruple slot passing frequencies are still not obvious on the time–frequency plane. HSST (*N* = 4) was then applied to the fault vibration signals of the wind turbine, with the corresponding results shown in Figure 16c. Owing to the presence of irrelevant signal components (e.g., noise) on the time–frequency plane, its energy aggregation degree needs to be further enhanced. Finally, the TFA results obtained by the ISTFT–ASST method proposed in this paper are shown in Figure 16d, from which the double and quadruple harmonics of the slot passing frequency can be clearly identified. Figure 17 shows that the 2nd and 4th harmonics of slot passing frequency are clearly identified under varying speed conditions.

**Figure 18:**
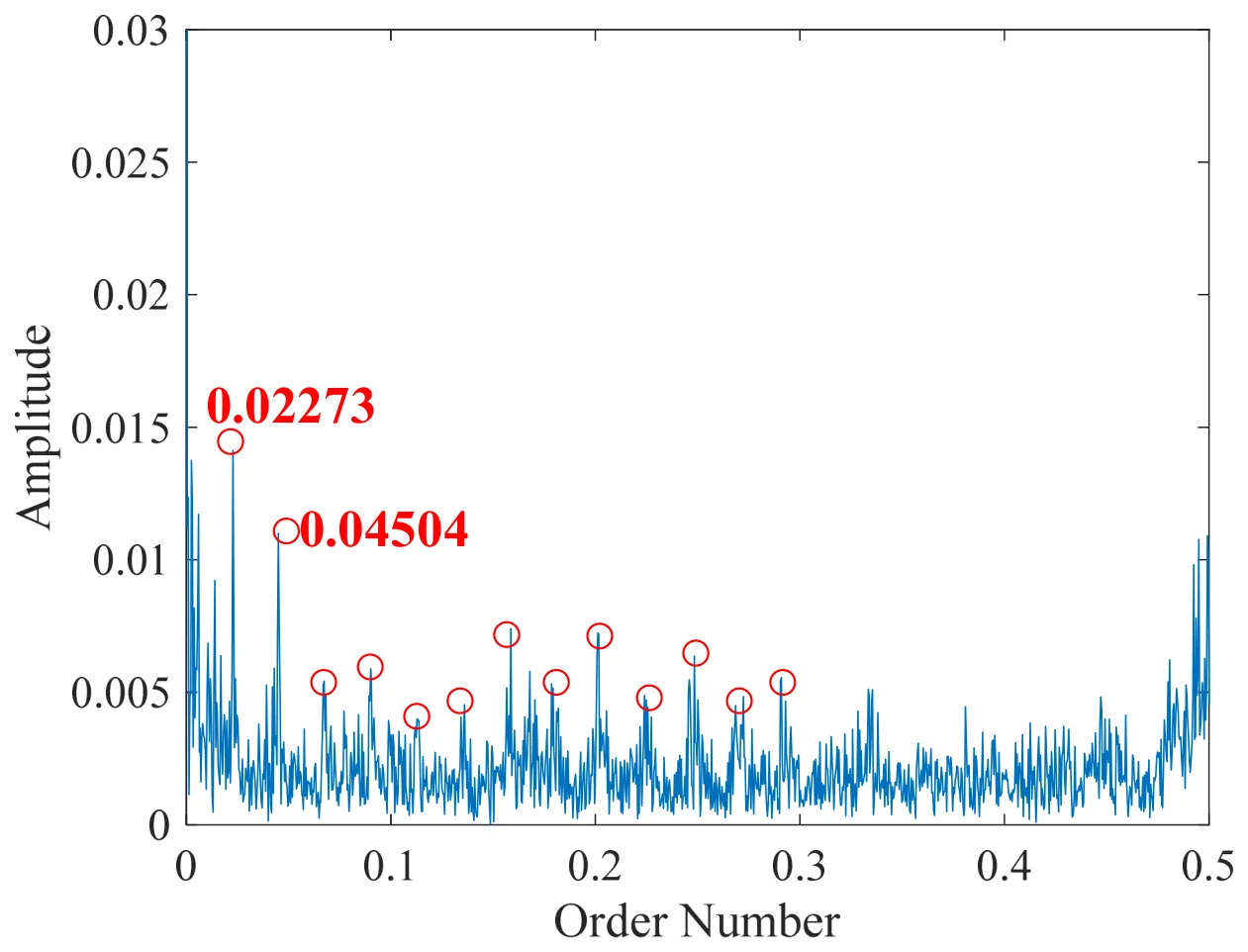
Order tracking envelope spectrum analysis results of wind turbine fault signal obtained by ISTFT-ASST.

Subsequently, order tracking envelope analysis was performed by taking the quadruple slot passing frequency (4F_s_) as the reference equivalent rotational frequency, with the results shown in Figure 18. The fault order 0.02273 and its second harmonic of 0.04504, which correspond to the inner race fault order, can be clearly observed in the figure. Meanwhile, the higher-order harmonics of the fault order, marked with red circles in the figure, can also be distinctly recognized. Therefore, an early inner race fault is diagnosed, which is consistent with the actual operating condition.

## 5. Conclusions

To address the core challenges of traditional TFA methods for wind turbine bearings under variable-speed operating conditions—namely the difficulty in accurately extracting fault features due to signals exhibiting multi-component non-stationarity, strong time-varying characteristics and susceptibility to noise interference—this paper proposes a novel ISTFT-ASST method. This method achieves precise estimation of the IF and IA of bearing fault signals under complex operating conditions, providing reliable technical support for early diagnosis and precise localization of wind turbine generator bearing faults, with competitive advantages over conventional FRI-STFT and other adaptive time–frequency solutions in terms of anti-noise performance and practical adaptability.

Specific conclusions are summarized as follows: (1) First, the proposed method overcomes the limitations of traditional fixed-window STFT by constructing a sparse observation model based on causal analysis windows. By establishing a relationship with the FRI model, the time–frequency representation is transformed into a convolution form between sparse Dirac combs and kernel functions. This design not only resolves the trade-off between time–frequency resolution caused by fixed windows but also establishes a structured theoretical basis for subsequent precise parameter estimation, thereby improving the quality and sparsity of the fundamental TFA. (2) Building upon the optimized STFT framework, this study integrates the time–frequency sharpening capability of the SST with the modal separation advantage of the SET. SST drives time–frequency coefficients toward true ridges, while SET applies normalized weighting to enhance target modes and suppress noise and cross-interference. This synergistic strategy compensates for the performance limitations of individual post-processing techniques, significantly improving the modal separation accuracy of strongly overlapping multi-component signals. (3) Drawing inspiration from the evaluation mechanism of local Rényi entropy for time–frequency clustering, a dynamic optimization method for time-varying window parameters is designed. By minimizing local Rényi entropy, the window width automatically adapts to local frequency variations in the signal. This algorithm addresses the challenge of manual parameter setting in traditional methods, which struggle to adapt to signals with rapidly changing frequencies, and balances estimation accuracy with real-time performance.

Based on the comprehensive validations including numerical simulation, laboratory bench test, and field wind turbine measurement, the proposed ISTFT-ASST method achieves consistent superior performance in time–frequency sparsity, anti-noise robustness, and instantaneous frequency tracking accuracy compared with conventional and state-of-the-art algorithms. Quantitative indices demonstrate that the method effectively suppresses noise interference, concentrates time–frequency energy, and tracks time-varying fault features stably under variable-speed conditions. Within wind turbine application scenarios, the proposed method also has definite applicable boundaries. It exhibits excellent adaptability and accuracy under moderate speed fluctuation and single–fault working conditions. However, under extreme rotational speed abrupt fluctuation or complex multi-component coupled composite fault scenarios, closely distributed time–frequency ridges cannot be completely separated, which may degrade feature extraction accuracy. In addition, the current experimental datasets lack labeled rolling-element fault samples, which further limits the comprehensive verification of the algorithm. In future research, we will further optimize the sparse reconstruction and modal weighting mechanism of the algorithm to enhance the separation capability of coupled fault components and improve robustness under extreme speed fluctuation conditions. Meanwhile, more rolling-element and compound fault samples will be collected for comprehensive verification to enhance the cross-scenario generalization capability and expand the engineering applicability of the proposed method for complex industrial fault diagnosis scenarios.

## Figures and Tables

**Table 1:** Comparison of Rényi entropy for different TFA methods.

**TFA Method**	**STFT**	**SST**	**SET**	**HSST**	**ISTFT-ASST**
Rényi Entropy	15.17	13.04	12.18	10.69	9.06

**Table 2:** Complete ablation study on numerical simulation analysis.

A (FRI Sparse Model)	B (SET Modal)	C (Adaptive Window Width)	Rényi Entropy	RMSE
No components				
×	×	×	15.17	0.82
Single components				
✓	×	×	14.26	0.73
×	✓	×	12.18	0.55
×	×	✓	12.31	0.59
Two components				
✓	✓	×	11.71	0.42
✓	×	✓	10.93	0.33
×	✓	✓	10.11	0.28
All components				
✓	✓	✓	9.06	0.19

**Table 3:** Rényi entropy of different TFA methods.

TFA Method	STFT	SST	SET	HSST	ISTFT-ASST
Rényi Entropy Value	20.93	18.64	15.15	13.71	9.35

**Table 4:** Comparison of Rényi entropy for several methods.

TF Methods	AMSCT	TSTPOW	ISTFT-ASST
Rényi entropy	18.6934	11.5388	9.35

**Table 5:** Mechanical characteristic frequencies of the wind turbine.

Serial Number	Component	Mechanical Characteristic	Characteristic Frequency (Hz)	Remarks
1	Main bearing BT2-8168/HA/VR602	Ball Pass Frequency of Inner race (BPFI)	20.88f_r_	f_r_ is the rotational frequency
2		Ball Pass Frequency of Outer race (BPFO)	18.12f_r_	
3		Ball Spin Frequency (BSF)	12.44f_r_	
4		Fundamental Train Frequency (FTF)	0.46f_r_	
5	Rear bearing NJ28/770ECMA/VAR567	Ball Pass Frequency of Inner race (BPFI)	25.74f_r_	
6		Ball Pass Frequency of Outer race (BPFO)	23.26f_r_	
7		Ball Spin Frequency (BSF)	19.72f_r_	
8		Fundamental Train Frequency (FTF)	0.475f_r_	

**Table 6:** Electrical characteristic frequencies of the wind turbine.

Serial Number	Electrical Characteristic	Characteristic Frequency (Hz)	Remarks
1	Feeding frequency (F_f_)	44f_r_	f_r_ is the rotational frequency
2	Pole passing frequency (F_p_)	44f_r_	
3	Ripple fundamental frequency (F_i_)	252f_r_	
4	Slot fundamental frequency (F_s_)	288f_r_	

## Data Availability

The data are not publicly available due to privacy or ethical restrictions.
